# Vitamin D status and functional parameters: A cross-sectional study in an older population

**DOI:** 10.1371/journal.pone.0201840

**Published:** 2018-08-21

**Authors:** J. Mendes, A. Santos, N. Borges, C. Afonso, P. Moreira, P. Padrão, R. Negrão, T. F. Amaral

**Affiliations:** 1 Department of Biomedicine, Biochemistry Unit, Faculty of Medicine, University of Porto, Porto, Portugal; 2 I3S - Institute for Research and Innovation in Health, University of Porto, Porto, Portugal; 3 Faculty of Nutrition and Food Sciences, University of Porto, Porto, Portugal; 4 CINTESIS - Centre for Health Technology and Services Research, Porto, Portugal; 5 EPIUnit, Institute of Public Health, University of Porto, Porto, Portugal; 6 The Research Centre in Physical Activity, Health and Leisure, University of Porto, Porto, Portugal; 7 System Integration and Process Automation Unit (UISPA), Mechanical Engineering Institute (IDMEC), Faculty of Engineering, University of Porto, Porto, Portugal; Medical University of Gdańsk, POLAND

## Abstract

The present study aims to clarify the association of vitamin D status with functionality, measured through gait speed and hand grip strength, in a sample of older adults, considering sex-specific characteristics and the potential confounding effect of lifestyle factors, disease, skin phenotype, season of blood collection and vitamin D supplementation. The Nutrition UP 65 cross-sectional study was conducted in a population-based sample of 1,425 older adults ≥ 65 years old. Serum levels of 25-hydroxyvitamin D were quantified through electrochemiluminescence immunoassay. Multinomial logistic regressions were carried out using quartiles of gait speed and of hand grip strength as dependent variables. Participants at risk of vitamin D inadequacy (30.0-49.9 nmol/L) and deficiency (< 30.0 nmol/L) presented higher adjusted odds ratio of the lowest values of gait speed and hand grip strength than those with adequate vitamin D levels (≥ 50.0 nmol/L). These associations were strongest among men at risk of vitamin D deficiency [adjusted odds ratio for the lowest quartile of gait speed = 3.24; 95% CI: 1.56–6.73 and for the lowest quartile of hand grip strength = 3.28; 95% CI: 1.47-7.31] than in women at risk of vitamin D deficiency [adjusted odds ratio for the lowest quartile of gait speed = 2.72; 95% CI: 1.37-5.41 and for the lowest quartile of hand grip strength = 1.56; 95% CI: 0.81–3.00]. In conclusion, in older adults, particularly in men, the risk of vitamin D deficiency was directly associated with the lowest values of gait speed and of hand grip strength. However, randomized controlled trials are needed to overcome the possibility of reverse causation and residual confounding. Present results emphasise the need for strategies to promote the reduction of the high prevalence of low vitamin D status among the Portuguese older adult population.

## Introduction

In the past 10 years, the improvement in health systems had as a consequence living longer [1]. However, among ageing world populations the loss of functional health has also been increasing, which has as consequences the poor quality of life, high health costs and increased mortality [1, 2].

Functional decline can be linked to socio-demographic and psychosocial aspects, lifestyle, nutritional status, disease and genetic predisposition [1]. In this context, vitamin D has an important role, controlling bone formation by interaction with osteoblasts, bone resorption through osteoclasts and regulating muscle function [3]. Vitamin D has also numerous targets in the immune, cardiovascular and nervous systems [3]. It is common to observe a higher risk of vitamin D inadequacy and deficiency among community-dwelling older adults [4].

Functional status is widely assessed through gait speed and hand grip strength which are objective functional measures of neuromuscular function and physical performance [5, 6]. Although the measures are correlated to each other, they are also indicators of distinct aspects of functionality [7, 8]. Gait speed is related to balance and relies mainly on actions of the lower extremity musculature [5], while hand grip strength reflects the strength of upper body structure [6, 8]. In older adults, these functional parameters are of particular interest because they are good predictors of functional disability, morbidity and mortality [7].

The association between serum vitamin D and physical functionality in older adults has been explored but some methodologic issues warrant deeper insight. The serum 25-hydroxyvitamin D (25(OH)D) concentrations were positively associated with gait speed in a meta-analysis that included twenty-two studies [9]. However, only the Timed Up and Go test was used to assess functionality in three studies [10–12] and among the remaining nineteen studies where gait speed was measured, the potential confounding effect of factors such as skin phenotype, season of blood collection and vitamin D supplementation was not analyzed in fourteen [13–26]. Regarding the five studies where at least one of the aforementioned potential confounders was considered, one study was restricted to women [27], one other to men [28], two included a mixed sample of adults and older adults [29, 30] and the last was restricted to the oldest old participants [31]. A more recent cross-sectional research including only non-physically active older adults, revealed a lack of association between serum 25(OH)D and gait speed [32].

The results concerning the association between serum 25(OH)D and hand grip strength are still controversial [33]. Indeed, no association of vitamin D with hand grip strength was observed in women aged ≥ 75 years through a cross-sectional study [34] and in individuals of both sexes aged ≥ 80 years among baseline results of the Belfrail study [31]. However, in both studies, more than 80% of participants presented vitamin D insufficiency, and the low amplitude of serum 25(OH)D values could have impaired the ability to identify a potential association between vitamin D and hand grip strength [31, 34].

Thus, questions remain regarding the strength of the association between serum 25(OH)D and physical functional status. Given the fact that both are potentially modifiable [1–3], the elucidation of their association among older adults remains of major relevance. The present study aims to clarify the association of serum 25(OH)D levels with both gait speed and hand grip strength in a population-based sample of older adults, considering sex-specific characteristics and the potential confounding effect of lifestyle factors, disease, skin phenotype, season of blood collection and vitamin D supplementation.

## Materials and methods

### Study design and sampling

The Nutrition UP 65 cross-sectional study was conducted in a cluster sample of 1,500 Portuguese older adults ≥ 65 years old, between December 2015 and June 2016. Information related to study design and data collection was previously described [35]. Descriptive data of the total sample for serum 25(OH)D [36], hand grip strength [37] and gait speed [38] were published.

A nationally representative sample of older adults in terms of sex, age, educational level and area of residence was formed through data from the 2011 census [35]. Nomenclature of Territorial Units for Statistical was used to determine regional areas [35]. A random, stratified and cluster sampling method was conducted. In each regional area, three or more town councils with > 250 inhabitants were randomly selected. The potential participants were contacted directly via town councils and parish centres. Eligibility criteria included having Portuguese nationality, current tax residence in Portugal, and being ≥ 65 years old. The presence of lower and/or upper limb deformities, and/or inability to perform gait speed or hand grip strength measurements, due to situations that led to not understanding the explanations and to not performing the techniques correctly, were set as exclusion criteria.

### Data collection and variable definition

Sex, date of birth and education composed demographic data. Level of education was based on the number of completed years of school and the following education categories were created: “no formal schooling”, “1 to 4 schooling years” and “> 4 years schooling years”.

Lifestyle parameters included data about alcoholic beverages consumption and physical activity. Participants were asked if they consumed alcoholic beverages and undertook physical activity during the previous seven days. Physical activity was assessed by the International Physical Activity Questionnaire–Short Form [39]. The time that participants spent walking or practicing some type of activity, within the domains of household, yard work activities, occupational activity, and leisure-time physical activity was estimated [39].

The Mini Mental State Examination (MMSE) was used to assess the cognitive performance [40]. The cut-off scores for cognitive impairment are as follows: individuals with no education, < 15 points; 1 to 11 years of years of school completed, < 22 points; and > 11 years of school completed, < 27 points [40]. For individuals identified as presenting cognitive impairment, reported data was provided by a family member close to the participant or a caregiver. Questions from the Portuguese National Health Survey 2005–2006 were used to collect data regarding the presence of chronic diseases [41].

Body weight, in kilograms, was measured with the participants wearing light clothes using a calibrated portable electronic scale (Seca 803, SECA GmbH, Hamburg, Germany), 0.1 kg resolution. Standing height was obtained with a calibrated stadiometer (Seca 213, SECA GmbH, Hamburg, Germany) with 0.1 cm resolution. In participants with visible kyphosis (n = 17, 1.2%), it was not possible to measure height with the stadiometer, and it was estimated indirectly from non-dominant hand length (in centimetres), measured with a calibrated calliper (Fervi C056, FERVI, 20 Vignola, Italy) with 0.1 cm resolution [35]. Body mass index (BMI) was calculated by the formula: weight [kg]/ (height [m])^2^ and classified according to the World Health Organization criteria (normal BMI ranged between 18.5 and 24.9 Kg/ m^2^; overweight ranged between 25.0 and 29.9 Kg/ m^2^ and a BMI ≥ 30.0 Kg/ m^2^ was indicator of obesity) [42].

### Functional parameters: Gait speed and hand grip strength

Gait speed was calculated dividing the distance walked 4.6 m on a flat and unobstructed path, by the time (in seconds) to walk that distance [43]. A visible tape was used to mark the distance on the floor. Each participant received the following instruction: “I will say ready, set, go. When I say go, walk at you normal and comfortable pace until I say stop” [43]. A stopwatch with a resolution of 0.01 s (School Electronic Stopwatch, Dive049, Topgim, Portugal) was used to register the walking time. Gait speed was categorized according to sex-specific quartiles whose classes were: in women: 1^st^ quartile: < 0.60 m/s; 2^nd^ quartile: [0.60–0.81 m/s[; 3^rd^ quartile [0.81–1.04 m/s [and 4^th^ quartile: ≥ 1.04 m/s and in men: 1^st^ quartile: < 0.74 m/s; 2^nd^ quartile: [0.74–0.94 m/s[; 3^rd^ quartile [0.94–1.19 m/s [and 4^th^ quartile: ≥ 1.19 m/s.

Hand grip strength data was obtained with a Jamar Plus^®^ + Digital Hand Dynamometer (Sammons Preston Inc., Bolingbrook, Illinois, USA) calibrated by the manufacturer, with a resolution of 0.1 Kgf. Measurements were carried out with the subject seated, shoulders adducted and neutrally rotated, elbow flexed at 90°, forearm in neutral and wrist between 0 and 30° of dorsiflexion, as recommended by the American Society of Hand Therapists [44]. Three measurements with one-minute pause between them were performed by each participant. The maximum value of three consecutive measurements in the non-dominant hand was registered. The dominant hand was used when the individual was unable to perform the measurement with the non-dominant hand, (n = 18, 1.3%). Hand grip strength was categorized according to sex-specific quartiles whose classes were: in women: 1^st^ quartile: < 14.5 Kgf; 2^nd^ quartile: [14.6–17.7 Kgf[; 3^rd^ quartile: [17.7–21.8 Kgf [and 4^th^ quartile: ≥ 21.8 Kgf, and in men: 1^st^ quartile: < 24.8 Kgf; 2^nd^ quartile: [24.8–30.4 Kgf[; 3^rd^ quartile: [30.4–36.9 Kgf [and 4^th^ quartile: ≥ 36.9 Kgf.

### Vitamin D

For each participant, a blood sample was collected and the collection date was registered. A certified laboratory, Labco Portugal, was responsible for blood collection and analyses. Qualified nurses collected the blood samples and 25(OH)D serum levels were measured with an electrochemiluminescence immunoassay. In this vitamin D total assay, vitamin D-binding protein was employed to capture both 25(OH)D3 and 25(OH)D2. Traceability was standardized against liquid chromatography (LC)—mass spectrometry (MS)/MS which in turn was traceable to the National Institute of Standards and Technology (NIST) (Roche Cobas Vitamin D total assay reagent kit, Roche Diagnostics, Mannheim, Germany). The same equipment was used for all analyses. Participants were classified as presenting adequate 25(OH)D levels (≥ 50.0 nmol/L), as being at risk of inadequacy (30.0–49.9 nmol/L) or as being at risk of deficiency (< 30.0 nmol/L), using the US Institute of Medicine (IOM) cut-off points [45]. According to the date of blood collection, participants were grouped into the following two categories: Autumn/Winter and Spring/Summer. Participants were also asked if they use vitamin D supplements.

Vitamin D synthesis is highly dependent on the concentration of melanin in the skin [46] and therefore skin phenotype was also evaluated using the Fitzpatrick classification [47] and regrouped into three categories: “pale white or white skin, blond or red hair, freckles”; “cream white skin or moderate brown skin” and “dark brown skin or black people”.

### Statistical analysis

Statistical analyses were conducted in 1,425 participants (95% of the total original dataset). Exclusions were due to missing gait speed or hand grip strength records in 37 (2.5%) cases and gait speed was also not evaluated in 27 (1.8%) participants with mobility restrictions. In 11 (0.7%) cases there were missing variables related to self-reported chronic diseases. A sensitivity analysis was carried out to evaluate the potential impact of exclusions in results. Compared to the participants included in the study (mean age of 74.9 ± 7.0 years), excluded individuals were older (mean age of 76.7 ± 7.9 years), *p* = 0.032, and no significant differences were found for all other studied variables.

Characteristics of the sample were presented stratified by quartiles of gait speed and of hand grip strength, in women and men. Categorical variables were summarized as counts and proportions and compared using the chi-square test. Kolmogorov-Smirnov test was used to examine the normality of variables distribution. Means and their standard deviation values were presented and were compared through ANOVA analysis. Medians and interquartile ranges (IQRs) were presented for variables without normal distribution and compared using the Mann-Whitney test.

Multinomial logistic regressions were performed for women and men, using quartiles of gait speed and of hand grip strength as dependent variables. Crude and adjusted odds ratios (OR) with 95% confidence intervals (CI) were estimated to quantify the associations of vitamin D status with each of the three quartiles of gait speed and of hand grip strength, having always the 4^th^ quartile (higher values of gait speed and hand grip strength) as the reference category. Associations were adjusted for age, education level, body mass index categories, alcoholic beverages consumption, physical activity, cognitive performance, number of chronic diseases, skin phenotype, season of blood collection and use of vitamin D supplements.

Results were considered significant when *p* < 0.05. All the statistical analysis was carried out with Statistical Package for Social Sciences for Windows (SPSS, version 24.0).

### Ethics

This study was conducted according to the guidelines of the Declaration of Helsinki. Potential participants were contacted by the interviewer, who provided information about the study purposes and methodology, and invited them to participate. In cases of acceptance, cognitive performance was assessed by the version of Mini Mental State Examination validated for the Portuguese population with cut-offs depending on the educational level [40]. Individuals without cognitive impairment were asked to read and sign a duplicated informed consent form. If the participant was deemed to be cognitively impaired, two representatives were asked to read and sign the duplicated informed consent form. And so, in this way, written informed consent was obtained from participants or their legally authorized representatives.

The study protocol was approved by the Ethics Committee of Social Sciences and Health from Faculty of Medicine of University of Porto, Portugal (PCEDCSS–FMUP 15/2015) and by the Portuguese National Commission of Data Protection (9427/2015).

## Results

The analyzed sample was composed of 58.5% of women with a mean of age of 75.4 ± 7.5 years and by 41.5% of men with a mean of age of 74.2 ± 6.7 years. In both sexes, statistically significant differences were found according to quartiles of gait speed and also of hand grip strength, regarding socio-demographic and lifestyle parameters, cognition status, the season of blood collection and the use of vitamin D supplementation (Tables 1 to 6).

**Table 1:** Socio-demographic and lifestyle parameters of women, according to quartiles of gait speed and of hand grip strength.

	Women (n = 834)									
	Gait speed (m/s)					Hand grip strength (Kgf)				
	1^st^ quartile < 0.60 (n = 207)	2^nd^ quartile [0.60–0.81[ (n = 210)	3^rd^ quartile [0.81–1.04[ (n = 205)	4^th^ quartile ≥ 1.04 (n = 212)	*p*	1^st^ quartile < 14.6 (n = 207)	2^nd^ quartile [14.6–17.7[ (n = 208)	3^rd^ quartile [17.7–21.8[ (n = 211)	4^th^ quartile ≥ 21.8 (n = 208)	*p*
**Socio-demographic parameters**										
Age (years), mean (SD)	80.2 (7.2)	76.6 (6.6)	73.2 (6.2)	71.5 (5.4)	< 0.001	79.5 (7.6)	76.5 (6.6)	74.3 (6.9)	71.3 (4.8)	< 0.001
Education (years), n (%)										
• no formal schooling	54 (26.1)	43 (20.5)	30 (14.6)	17 (8.0)	< 0.001	41 (19.8)	37 (17.8)	43 (20.4)	23 (11.1)	0.015
• 1–4 schooling years	139 (67.1)	143 (68.1)	134 (65.4)	157 (74.1)		149 (72.0)	142 (68.3)	133 (63.0)	149 (71.6)	
• ˃ 4 schooling years	14 (6.8)	24 (11.4)	41 (20.0)	38 (17.9)		17 (8.2)	29 (13.9)	35 (16.6)	36 (17.3)	
**Lifestyle parameters**										
Alcoholic beverages consumption ^1^, n (%)										
• no	145 (70.4)	147 (70.3)	124 (60.5)	96 (45.3)	< 0.001	157 (75.8)	142 (68.6)	121 (57.6)	92 (44.2)	< 0.001
• yes	61 (29.6)	62 (29.7)	81 (39.5)	116 (54.7)		50 (24.2)	65 (31.4)	89 (42.4)	116 (55.8)	
Physical activity (hours/day), mean (SD)	0.9 (0.5)	1.1 (0.9)	2.0 (1.7)	2.4 (2.1)	< 0.001	1.1 (0.7)	1.3 (1.1)	1.7 (1.4)	2.3 (2.1)	< 0.001

^**1**^ Information was not reported by two participants (0.2%). Values shown in parentheses refer to SD or to column percentage number; *p* values are relative to the differences between quartiles. *Abbreviation*: SD, standard deviation.

**Table 2:** Cognition, subjective health and nutritional parameters of women, according to quartiles of gait speed and of hand grip strength.

	Women (n = 834)									
	Gait speed (m/s)					Hand grip strength (Kgf)				
	1^st^ quartile < 0.60 (n = 207)	2^nd^ quartile [0.60–0.81[ (n = 210)	3^rd^ quartile [0.81–1.04[ (n = 205)	4^th^ quartile ≥ 1.04 (n = 212)	*p*	1^st^ quartile < 14.6 (n = 207)	2^nd^ quartile [14.6–17.7[ (n = 208)	3^rd^ quartile [17.7–21.8[ (n = 211)	4^th^ quartile ≥ 21.8 (n = 208)	*p*
**Cognition and subjective health**										
Mini-Mental State Examination ^1^, n (%)										
• without cognitive impairment	182 (87.9)	196 (93.3)	194 (94.6)	210 (99.1)	< 0.001	175 (84.5)	193 (92.8)	199 (94.3)	206 (99.0)	< 0.001
• with cognitive impairment	25 (12.1)	14 (6.7)	11 (5.4)	2 (0.9)		32 (15.5)	15 (7.2)	12 (5.7)	2 (1.0)	
Number of chronic diseases, mean (SD)	4.6 (1.9)	4.3 (2.3)	4.3 (2.0)	3.9 (2.1)	0.031	4.6 (2.1)	4.5 (2.1)	4.2 (2.2)	3.8 (1.9)	0.005
**Nutritional parameters**										
Body mass index ^2^, n (%)										
• normal	22 (10.6)	25 (11.9)	29 (14.1)	38 (17.9)	0.001	34 (16.4)	27 (13.0)	28 (13.3)	25 (12.0)	0.156
• overweight	82 (39.6)	68 (32.4)	85 (41.5)	103 (48.6)		77 (37.2)	75 (36.1)	90 (42.6)	96 (46.2)	
• obesity	103 (49.8)	117 (55.7)	91 (44.4)	71 (33.5)		96 (46.4)	106 (50.9)	93 (44.1)	87 (41.8)	
Vitamin D status, n (%)										
• adequacy (≥ 50.0 nmol/L 25(OH)D)	31 (15.0)	54 (25.7)	64 (31.2)	81 (38.2)	< 0.001	39 (18.8)	52 (25.0)	62 (29.4)	63 (30.3)	< 0.001
• at risk of inadequacy (30.0–49.9 nmol/L 25(OH)D)	40 (19.3)	54 (25.7)	65 (31.7)	67 (31.6)		35 (16.9)	66 (31.7)	57 (27.0)	68 (32.7)	
• at risk of deficiency (< 30.0 nmol/L 25(OH)D)	136 (65.7)	102 (48.6)	76 (37.1)	64 (30.2)		133 (64.3)	90 (43.3)	92 (43.6)	77 (37.0)	

^**1**^ Participants were considered to have no cognitive impairment with a score above 15 if they had no formal schooling, above 22 if they had ≤ 11 years of education and above 27 if they had > 11 years of education [40].

^**2**^ Using World Health Organization cut-off points [42]. Values shown in parentheses refer to SD or to column percentage number; *p* values are relative to the differences between quartiles. *Abbreviation*: 25(OH)D, 25-hydroxyvitamin D; SD, standard deviation.

**Table 3:** Skin phenotype, season of blood collection and use of vitamin D supplements in women, according to quartiles of gait speed and of hand grip strength.

	Women (n = 834)									
	Gait speed (m/s)					Hand grip strength (Kgf)				
	1^st^ quartile < 0.60 (n = 207)	2^nd^ quartile [0.60–0.81[ (n = 210)	3^rd^ quartile [0.81–1.04[ (n = 205)	4^th^ quartile ≥ 1.04 (n = 212)	*p*	1^st^ quartile < 14.6 (n = 207)	2^nd^ quartile [14.6–17.7[ (n = 208)	3^rd^ quartile [17.7–21.8[ (n = 211)	4^th^ quartile ≥ 21.8 (n = 208)	*p*
**Skin phenotype** ^1^, **n (%)**										
Pale white or white skin, blond or red hair, freckles	57 (27.5)	42 (20.2)	44 (21.5)	50 (23.6)	0.336	55 (26.6)	49 (23.7)	51 (24.2)	38 (18.4)	0.127
Cream white skin or moderate brown skin	139 (67.2)	151 (72.6)	153 (74.6)	154 (72.6)		144 (69.5)	142 (68.6)	154 (73.0)	157 (75.8)	
Dark brown skin or black people	11 (a5.3)	15 (7.2)	8 (3.9)	8 (3.8)		8 (3.9)	16 (7.7)	6 (2.8)	12 (5.8)	
**Season of blood sample collection, n (%)**										
Autumn/ Winter	167 (80.7)	145 (69.0)	120 (58.5)	107 (50.5)	< 0.001	144 (69.6)	143 (68.8)	139 (65.9)	113 (54.3)	0.004
Spring/ Summer	40 (19.3)	65 (31.0)	85 (41.5)	105 (49.5)		63 (30.4)	65 (31.2)	72 (34.1)	95 (45.7)	
**Use of supplements with vitamin D, n (%)**										
No	162 (78.3)	179 (85.3)	170 (82.9)	190 (89.6)	< 0.001	169 (81.6)	166 (79.8)	184 (87.2)	182 (87.5)	< 0.001
Yes	14 (6.8)	15 (7.1)	17 (8.3)	19 (9.0)		9 (4.3)	19 (9.1)	16 (7.6)	21 (10.1)	
Unknown composition or use	31 (14.9)	16 (7.6)	18 (8.8)	3 (1.4)		29 (14.1)	23 (11.1)	11 (5.2)	5 (2.4)	

^**1**^Information was not registered for two participants (0.2%). Values shown in parentheses refer to column percentage number; *p* values are relative to the differences between quartiles.

**Table 4:** Socio-demographic and lifestyle parameters of men, according to quartiles of gait speed and of hand grip strength.

	Men (n = 591)									
	Gait speed (m/s)					Hand grip strength (Kgf)				
	1^st^ quartile < 0.74 (n = 147)	2^nd^ quartile [0.74–0.94[ (n = 147)	3^rd^ quartile [0.94–1.19[ (n = 147)	4^th^ quartile ≥ 1.19 (n = 150)	*p*	1^st^ quartile < 24.8 (n = 148)	2^nd^ quartile [24.8–30.4[ (n = 148)	3^rd^ quartile [30.4–36.9[ (n = 147)	4^th^ quartile ≥ 36.9 (n = 148)	*p*
**Socio-demographic parameters**										
Age (years), mean (SD)	78.2 (7.6)	74.6 (6.1)	72.4 (5.5)	71.6 (5.4)	< 0.001	77.8 (7.6)	75.7 (6.6)	73.0 (5.3)	70.2 (4.4)	< 0.001
Education (years), n (%)										
• no formal schooling	26 (17.7)	14 (9.5)	6 (4.1)	5 (3.4)	< 0.001	19 (12.8)	21 (14.2)	8 (5.5)	3 (2.0)	< 0.001
• 1–4 schooling years	100 (68.0)	103 (70.1)	101 (68.7)	104 (69.3)		105 (71.0)	101 (68.2)	104 (70.7)	98 (66.2)	
• ˃ 4 schooling years	21 (14.3)	30 (20.4)	40 (27.2)	41 (27.3)		24 (16.2)	26 (17.6)	35 (23.8)	47 (31.8)	
**Lifestyle parameters**										
Alcoholic beverages consumption, n (%)										
• no	65 (44.2)	46 (31.3)	35 (23.8)	25 (16.7)	< 0.001	70 (47.3)	54 (36.5)	28 (19.0)	19 (12.8)	< 0.001
• yes	82 (55.8)	101 (68.7)	112 (76.2)	125 (83.3)		78 (52.7)	94 (63.5)	119 (81.0)	129 (87.2)	
Physical activity (hours/day), mean (SD)	1.2 (0.9)	1.9 (1.4)	2.1 (1.9)	2.5 (2.1)	< 0.001	1.3 (1.1)	1.8 (1.3)	2.2 (2.1)	2.5 (2.2)	< 0.001

Values shown in parentheses refer to SD or to column percentage number; *p* values are relative to the differences between quartiles. *Abbreviation*: SD, standard deviation.

**Table 5:** Cognition, subjective health and nutritional parameters of men, according to quartiles of gait speed and of hand grip strength.

	Men (n = 591)									
	Gait speed (m/s)					Hand grip strength (Kgf)				
	1^st^ quartile < 0.74 (n = 147)	2^nd^ quartile [0.74–0.94[ (n = 147)	3^rd^ quartile [0.94–1.19[ (n = 147)	4^th^ quartile ≥ 1.19 (n = 150)	*p*	1^st^ quartile < 24.8 (n = 148)	2^nd^ quartile [24.8–30.4[ (n = 148)	3^rd^ quartile [30.4–36.9[ (n = 147)	4^th^ quartile ≥ 36.9 (n = 148)	*p*
**Cognition and subjective health**										
Mini-Mental State Examination ^1^, n (%)										
• without cognitive impairment	132 (89.8)	140 (95.2)	145 (98.6)	148 (98.7)	< 0.001	132 (89.2)	144 (97.3)	145 (98.6)	144 (97.3)	< 0.001
• with cognitive impairment	15 (10.2)	7 (4.8)	2 (1.4)	2 (1.3)		16 (10.8)	4 (2.7)	2 (1.4)	4 (2.7)	
Number of chronic diseases, mean (SD)	3.2 (2.0)	3.1 (1.9)	2.9 (1.7)	3.1 (1.8)	0.570	3.0 (1.9)	3.3 (2.0)	3.0 (1.6)	3.0 (1.8)	0.317
**Nutritional parameters**										
Body mass index ^2^, n (%)										
• normal	30 (20.4)	34 (23.1)	18 (12.3)	27 (18.0)	0.281	29 (19.6)	31 (20.9)	29 (19.7)	20 (13.5)	0.210
• overweight	63 (42.9)	72 (49.0)	84 (57.1)	76 (50.7)		73 (49.3)	66 (44.6)	82 (55.8)	74 (50.0)	
• obesity	54 (36.7)	41 (27.9)	45 (30.6)	47 (31.3)		46 (31.1)	51 (34.5)	36 (24.5)	54 (36.5)	
Vitamin D status, n (%)										
• adequacy (≥ 50.0 nmol/L 25(OH)D)	30 (20.4)	58 (39.4)	55 (37.4)	80 (53.4)	< 0.001	36 (24.3)	51 (34.4)	62 (42.2)	74 (50.0)	< 0.001
• at risk of inadequacy (30.0–49.9 nmol/L 25(OH)D)	41 (27.9)	46 (31.3)	61 (41.5)	41 (27.3)		49 (33.1)	39 (26.4)	48 (32.6)	53 (35.8)	
• at risk of deficiency (< 30.0 nmol/L 25(OH)D)	76 (51.7)	43 (29.3)	31 (21.1)	29 (19.3)		63 (42.6)	58 (39.2)	37 (25.2)	21 (14.2)	

^**1**^ Participants were considered to have no cognitive impairment with a score above 15 if they had no formal schooling, above 22 if they had ≤ 11 years of education and above 27 if they had > 11 years of education [40].

^**2**^ Using World Health Organization cut-off points [42]. Values shown in parentheses refer to SD or to column percentage number; *p* values are relative to the differences between quartiles. *Abbreviation*: 25(OH)D, 25-hydroxyvitamin D; SD, standard deviation.

**Table 6:** Skin phenotype, season of blood collection and use of vitamin D supplements in men, according to quartiles of gait speed and of hand grip strength.

	Men (n = 591)									
	Gait speed (m/s)					Hand grip strength (Kgf)				
	1^st^ quartile < 0.74 (n = 147)	2^nd^ quartile [0.74–0.94[ (n = 147)	3^rd^ quartile [0.94–1.19[ (n = 147)	4^th^ quartile ≥ 1.19 (n = 150)	*p*	1^st^ quartile < 24.8 (n = 148)	2^nd^ quartile [24.8–30.4[ (n = 148)	3^rd^ quartile [30.4–36.9[ (n = 147)	4^th^ quartile ≥ 36.9 (n = 148)	*p*
**Skin phenotype** ^1^, **n (%)**										
Pale white or white skin, blond or red hair, freckles	27 (18.4)	20 (13.6)	38 (26.0)	19 (12.7)	0.072	30 (20.3)	25 (17.0)	21 (14.3)	28 (18.9)	0.742
Cream white skin or moderate brown skin	112 (76.2)	117 (79.6)	101 (69.2)	121 (80.7)		109 (73.6)	116 (78.9)	115 (78.2)	111 (75.0)	
Dark brown skin or black people	8 (5.4)	10 (6.8)	7 (4.8)	10 (6.6)		9 (6.1)	6 (4.1)	11 (7.5)	9 (6.1)	
**Season of blood sample collection** ^2^, **n (%)**										
Autumn/ Winter	71 (48.3)	59 (40.1)	40 (27.4)	34 (22.7)	< 0.001	66 (44.9)	60 (40.5)	49 (33.3)	34 (23.0)	< 0.001
Spring/ Summer	76 (51.7)	88 (59.9)	106 (72.6)	116 (77.3)		81 (55.1)	88 (59.5)	98 (66.7)	114 (77.0)	
**Use of supplements with vitamin D, n (%)**										
No	128 (87.1)	130 (88.4)	139 (94.6)	147 (98.0)	0.003	127 (85.8)	130 (87.8)	141 (95.9)	146 (98.6)	< 0.001
Yes	2 (1.4)	7 (4.8)	3 (2.0)	3 (2.0)		8 (5.4)	4 (2.7)	2 (1.4)	1 (0.7)	
Unknown composition or use	17 (11.5)	10 (6.8)	5 (3.4)	0 (0.0)		13 (8.8)	14 (9.5)	4 (2.7)	1 (0.7)	

^**1**^ Information was not registered for one participant (0.2%).

^**2**^ Information was not reported by one participant (0.2%). Values shown in parentheses refer to column percentage number; *p* values are relative to the differences between quartiles.

Participants who presented lower gait speed and hand grip strength values were older, a significant proportion did not have formal schooling, did not drink alcoholic beverages and spent less physically active hours per day, compared to participants who presented higher gait speed and hand grip strength values (Tables 1 and 4).

Women presented lower mean values of gait speed (0.8 ± 0.3 m/s), than men (0.9 ± 0.3 m/s). Women also presented lower hand grip strength values than men, with a mean of 18.2 ± 5.3 Kgf *vs* 30.8 ± 8.9 Kgf, respectively.

The median value of serum 25(OH)D was 39.9 nmol/L (IQR: 33.9 nmol/L) in women and 42.8 nmol/L (IQR: 36.0 nmol/L) in men (*p* < 0.001). According to IOM cut-off points, 45.3% of women and 30.3% of men were at risk of vitamin D deficiency (< 30.0 nmol/L). Adequacy levels of vitamin D (≥ 50.0 nmol/L) were observed in 27.6% of women and in 37.7% of men.

**Fig 1:**
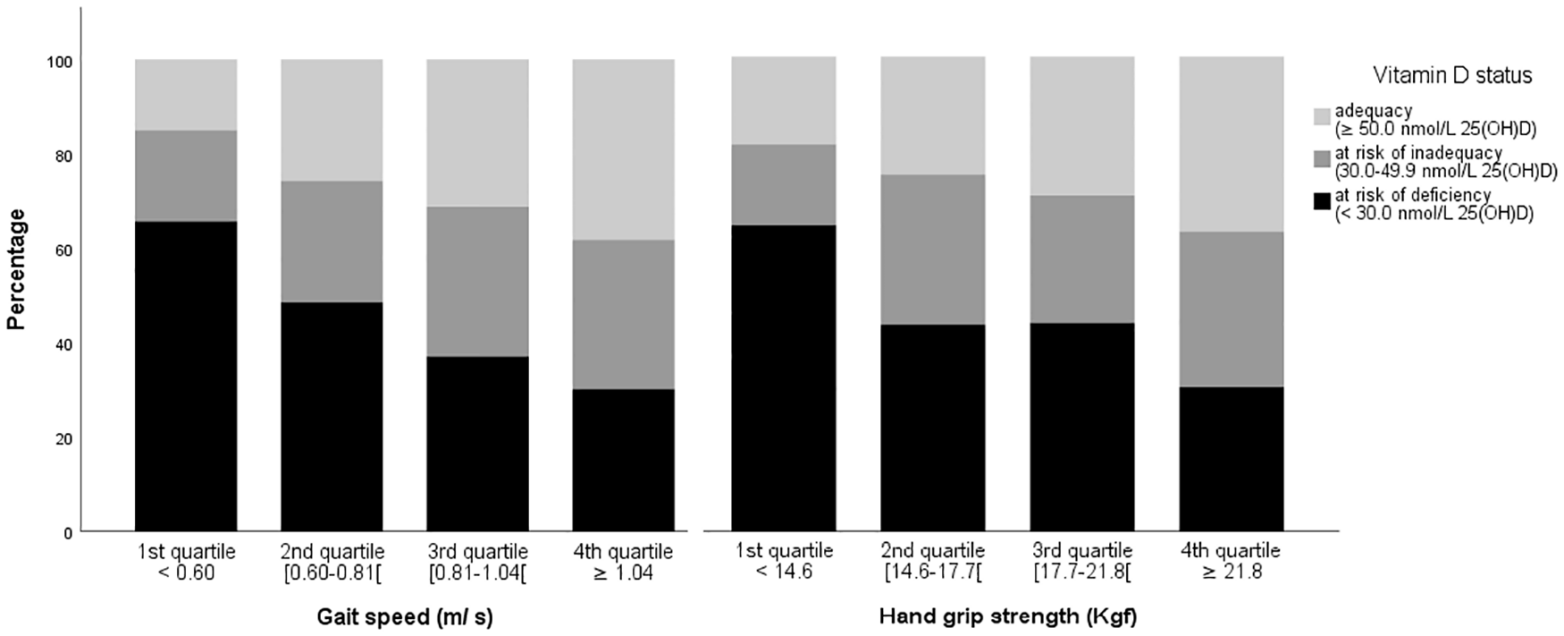
Women. Proportions of cases according to vitamin D status within quartiles of gait speed and of hand grip strength, in women.

**Fig 2:**
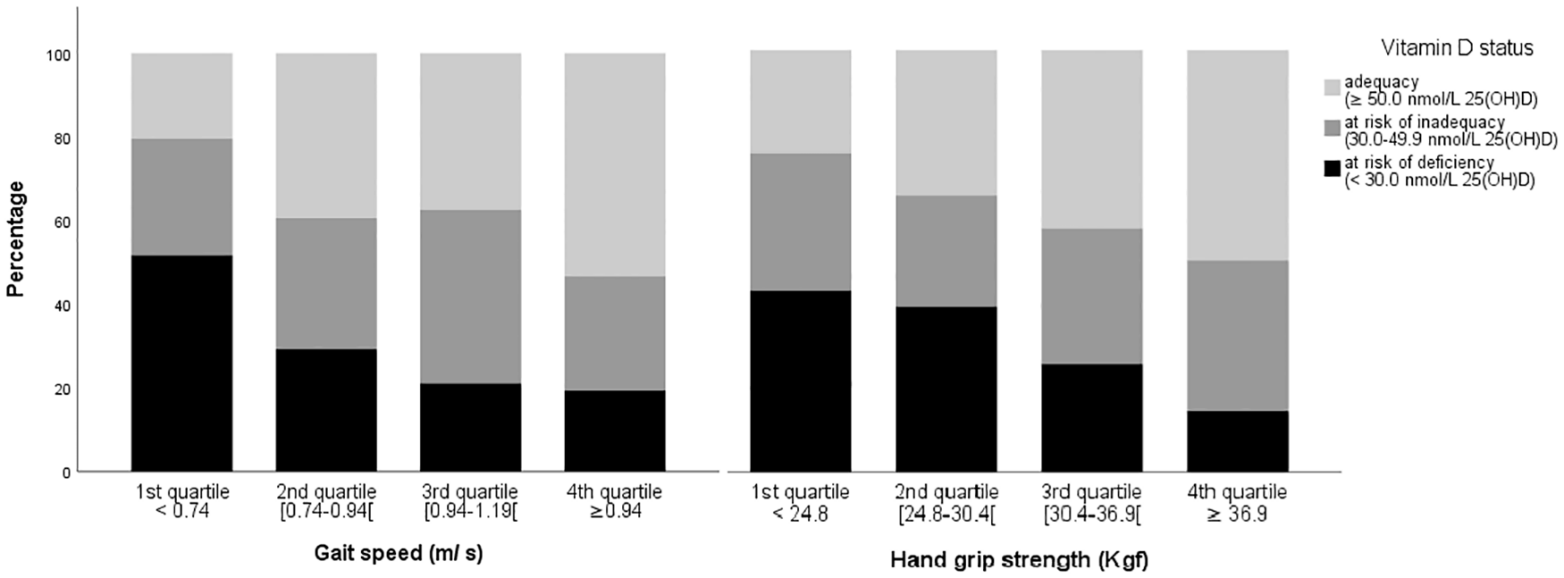
Men. Proportions of cases according to vitamin D status within quartiles of gait speed and of hand grip strength, in men.

In both sexes, the proportion of participants at risk of vitamin D deficiency decreased with increasing quartiles values of gait speed and hand grip strength (Figs 1 and 2).

Women at risk of vitamin D deficiency had higher adjusted OR of the lowest gait speed values (1^st^ quartile) [*vs*. presenting the highest gait speed values (4^th^ quartile)] than women with adequate vitamin D levels (adjusted OR for 1^st^ quartile of gait speed = 2.72; 95% CI: 1.37–5.41). However, adjusted OR for lower hand grip strength values according to vitamin D status were not statistically significant (Table 7).

**Table 7:** Multinomial logistic regressions of association between vitamin D status and quartiles of gait speed and of hand grip strength, in women.

	**Gait speed**					
	**1**^**st**^ **quartile** < 0.60 m/s		**2**^**nd**^ **quartile** [0.60–0.81 [m/s		**3**^**rd**^ **quartile** [0.81–1.04 [m/s	
	Crude OR (95% CI)	Adjusted ^1^ OR (95% CI)	Crude OR (95% CI)	Adjusted ^1^ OR (95% CI)	Crude OR (95% CI)	Adjusted ^1^ OR (95% CI)
**Vitamin D status**						
**Adequacy** ≥50.0 nmol/L 25(OH)D	1.00	1.00	1.00	1.00	1.00	1.00
**At risk of inadequacy** 30.0–49.9 nmol/L 25(OH)D	1.56 (0.88–2.76)	1.03 (0.49–2.14)	1.21 (0.74–1.99)	1.09 (0.52–1.73)	1.23 (0.77–1.97)	1.03 (0.61–1.76)
**At risk of deficiency** <30.0 nmol/L 25(OH)D	5.55** (3.34–9.24)	2.72* (1.37–5.41)	2.39** (1.50–3.81)	1.52 (0.84–2.77)	1.50 (0.94–2.40)	1.14 (0.65–2.01)
	**Hand grip strength**					
	**1**^**st**^ **quartile** < 14.6 Kgf		**2**^**nd**^ **quartile** [14.6–17.7 [Kgf		**3**^**rd**^ **quartile** [17.7–21.8 [kgf	
	Crude OR (95% CI)	Adjusted ^1^ OR (95% CI)	Crude OR (95% CI)	Adjusted ^1^ OR (95% CI)	Crude OR (95% CI)	Adjusted ^1^ OR (95% CI)
**Vitamin D status**						
**Adequacy** ≥50.0 nmol/L 25(OH)D	1.00	1.00	1.00	1.00	1.00	1.00
**At risk of inadequacy** 30.0–49.9 nmol/L 25(OH)D	1.02 (0.58–1.78)	1.04 (0.24–1.09)	1.44 (0.88–2.34)	1.07 (0.41–1.33)	1.04 (0.64–1.69)	1.05 (0.31–1.07)
**At risk of deficiency** <30.0 nmol/L 25(OH)D	4.17** (2.56–6.79)	1.56 (0.81–3.00)	2.12** (1.31–3.41)	1.09 (0.52–1.84)	1.81* (1.14–2.88)	1.11 (0.62–1.99)

Reference categories: the 4^th^ quartile of gait speed (≥ 1.04 m/s) and the 4^th^ quartile of hand grip strength (≥ 21.8 Kgf) are the reference categories and therefore they are not presented in the table.

* *p* < 0.05 and ** *p* < 0.001: *p* values for multinomial logistic regression analyses.

^1^ Adjusted for age (continuous), education (categorical), body mass index (categorical), alcoholic beverages consumption (dichotomous), physical activity (continuous), cognitive performance (dichotomous), number of chronic diseases (continuous), skin phenotype (categorical), season of blood sample collection (dichotomous) and use of vitamin D supplements (categorical). *Abbreviations*: 25(OH)D, 25-hydroxyvitamin D; CI, confidence intervals; OR, odds ratio.

For the men’s group, the associations were in the same direction as those identified for women but were stronger (adjusted OR for 1^st^ quartile of gait speed = 3.24; 95% CI: 1.56–6.73). Men at risk of vitamin D deficiency also had higher adjusted OR for presenting the lowest hand grip strength values (adjusted OR for 1^st^ quartile of hand grip strength = 3.28; 95% CI: 1.47–7.31) (Table 8). In addition, men at risk of vitamin D deficiency also had a significant higher adjusted OR for gait speed and hand grip strength values in 2^nd^ quartile [*vs*. 4^th^ quartile of gait speed and of hand grip strength] than men with adequate vitamin D levels (Table 8).

**Table 8:** Multinomial logistic regressions of association between vitamin D status and quartiles of gait speed and of hand grip strength, in men.

	**Gait speed**					
	**1**^**st**^ **quartile** < 0.74 m/s		**2**^**nd**^ **quartile** [0.74–0.94 [m/s		**3**^**rd**^ **quartile** [0.94–1.19 [m/s	
	Crude OR (95% CI)	Adjusted ^1^ OR (95% CI)	Crude OR (95% CI)	Adjusted ^1^ OR (95% CI)	Crude OR (95% CI)	Adjusted ^1^ OR (95% CI)
**Vitamin D status**						
**Adequacy** ≥50.0 nmol/L 25(OH)D	1.00	1.00	1.00	1.00	1.00	1.00
**At risk of inadequacy** 30.0–49.9 nmol/L 25(OH)D	2.67** (1.46–4.87)	1.73 (0.85–3.50)	1.55 (0.90–2.66)	1.54 (0.77–3.08)	1.56 (0.84–2.87)	1.35 (0.72–2.51)
**At risk of deficiency** <30.0 nmol/L 25(OH)D	6.99** (3.84–12.72)	3.24** (1.56–6.73)	2.05* (1.15–3.65)	2.07* (1.16–3.68)	2.16* (1.28–3.65)	1.32 (0.65–2.68)
	**Hand grip strength**					
	**1**^**st**^ **quartile** < 24.8 Kgf		**2**^**nd**^ **quartile** [24.8–30.4 [Kgf		**3**^**rd**^ **quartile** [30.4–36.9 [Kgf	
	Crude OR (95% CI)	Adjusted ^1^ OR (95% CI)	Crude OR (95% CI)	Adjusted ^1^ OR (95% CI)	Crude OR (95% CI)	Adjusted ^1^ OR (95% CI)
**Vitamin D status**						
**Adequacy** ≥50.0 nmol/L 25(OH)D	1.00	1.00	1.00	1.00	1.00	1.00
**At risk of inadequacy** 30.0–49.9 nmol/L 25(OH)D	1.90* (1.09–3.32)	1.13 (0.56–2.28)	1.07 (0.62–1.84)	1.08 (0.46–1.62)	1.08 (0.65–1.81)	1.07 (0.59–1.93)
**At risk of deficiency** <30.0 nmol/L 25(OH)D	6.17** (3.27–11.63)	3.28* (1.47–7.31)	4.00** (2.17–7.40)	2.17* (1.02–4.63)	2.10* (1.12–3.96)	1.84 (0.87–3.90)

Reference categories: the 4^th^ quartile of gait speed (≥ 1.19 m/s) and the 4^th^ quartile of handgrip strength (≥ 36.9 Kgf) are the reference categories and therefore they are not presented in the table.

* *p* < 0.05 and ** *p* < 0.001: *p* values for multinomial logistic regression analyses.

^1^Adjusted for age (continuous), education (categorical), body mass index (categorical), alcoholic beverages consumption (dichotomous), physical activity (continuous), cognitive performance (dichotomous), number of chronic diseases (continuous), skin phenotype (categorical), season of blood sample collection (dichotomous) and use of vitamin D supplements (categorical). *Abbreviations*: 25(OH)D, 25-hydroxyvitamin D; CI, confidence intervals; OR, odds ratio.

## Discussion

According to the present results, older adults at risk of vitamin D inadequacy and deficiency presented lower functional values of gait speed and hand grip strength, than those with adequate vitamin D levels. In fact, previous studies also indicated an association between low levels of vitamin D and low gait speed [9], but have revealed controversial results regarding the relation between vitamin D and hand grip strength [33].

In this regard, the present data contributed to clarify the association between vitamin D and functional parameters, overcoming methodological issues from previous research. Firstly, the association was quantified through both parameters in the same sample, hand grip strength and gait speed, providing more complete information of functional status of the studied older population. Secondly, the results were adjusted for a comprehensive set of potential confounders, such as supplementation use, skin phenotype and season of blood collection, which were not always considered in other studies [13–26]. The consideration of these factors is necessary because supplementation may change the effect of baseline vitamin D levels on functional status, and these levels are also affected by the amount of skin pigment and by the season of the year [36, 46, 48, 49]. The results of the association between vitamin D and functional status may also be affected by the amplitude of values available for both variables and also by the cut-off points used [9]. Although this study was conducted in a sample restricted to older adults, a wide range of both functional indicators and vitamin D values distribution is available, allowing the assess their association. Moreover, recommended cut-offs were used [45] to permit future comparisons.

In physiological terms, this association between vitamin D and functional parameters can be explained because bone and muscle are significantly affected by 25(OH)D serum concentration. In bone, vitamin D stimulates osteoclasts turnover and protects osteoblasts from apoptosis [50]. Vitamin D regulates several genes transcription and signaling pathways, modulating calcium and phosphorus homeostasis, as well as the proliferation and differentiation of muscle cells [51]. It maintains the function of type II fibers, preserving muscle strength [52]. Severe 25(OH)D deficiency (< 25 nmol/L) has been linked to myopathy and muscle pain [53], and this can also explain the present association between low vitamin D values and low hand grip strength.

Other physiological effects of vitamin D can also be responsible for the results herein presented. Vitamin D also benefits cognitive and neuromuscular function and low levels of vitamin D are associated with a reduced neuronal function in the caudal primary motor cortex, contributing to explain the pathophysiology of gait disorders in older adults [54]. Indeed, in combination with physical exercise, vitamin D supplementation reduces the risk of falls [55] which are among the main causes of injury that lead to death in the older population [56, 57]. It is known that slow gait speed leads to instability and falls, although this effect is offset by the related decrease in step length [58]. Indeed, gait speed measurement has shown to be a strong predictor of falls [59]. The risk of falls is also associated with low muscle strength, especially in the lower extremity [60], but the hand grip strength measurement provided added value in the identification of community-dwelling older adults at risk of recurrent falls [61].

The inadequacy or deficiency of vitamin D may also be a consequence of low gait speed or hand grip strength values. When impaired physical performance is associated with less outdoor physical activity there is a consequent reduction in sunlight exposure, causing hypovitaminosis D [62].

The associations found in the present study were stronger in men than in women. A possible explanation for this difference may be the higher range of vitamin D values observed among men who also presented higher serum 25(OH)D levels than women, similarly to what was reported by Toffanello et al. [21]. Another possible explanation is that sex specific differences found in vitamin D levels can be a consequence of the higher proportion of obesity in women (45.8% of obese women *vs* 31.6% of obese men). Indeed, it was described that a high proportion of adipose tissue leads to a sequestration of vitamin D in this tissue and a decrease in its bioavailability [63]. Moreover, a recent study suggests that genetic variability in a region of the vitamin D receptor may be an important factor influencing adiposity [64].

In the total sample, a very high proportion of participants were found at risk of vitamin D deficiency (39.1%), even though IOM cut-off points were used for vitamin D categorization [45]. The IOM cut-off points are more conservative (risk of deficiency < 30.0 nmol/L 25(OH)D) [45] than those recommended by the Endocrine Society Clinical Guidelines (ESCG) (deficiency < 50.0 nmol/L 25(OH)D) [65]. If these ESCG guidelines were applied, more unbalanced groups would have been created, because more than half of our sample would present vitamin D deficiency. In addition, it is relevant to note that ESCG recommends that serum 25(OH)D concentration should surpass 75 nmol/L to maximize bone health and muscle function [65], but only 12.1% of our sample reached this value. This fact contributes to explain the low physical functionality of this sample of older adults.

The quantification of the association of vitamin D levels with gait speed and hand grip strength, considering a wide range of potential confounders, can be referred as a strength of the present study. The identified associations have implications not only for physical functioning but also on general health, once gait speed has been reported as an additional vital sign to be considered in the geriatric assessment [66] and hand grip strength has demonstrated to reflect overall muscle strength [67]. Moreover, in a recent study that reported over 15 years follow up of an older population, the adjusted hazard ratio of death ranged 1.43 to 2.07 in participants who presented functional impairment, demonstrating the impact of the physical functionality on mortality [68].

The interest of carrying out this study in Portugal was due to the fact that Portugal had the third highest aging index in Europe in 2016, after Italy and Germany, justifying the urgency in the prevention of the loss of independence among older adults [69]. Aging index is defined by the ratio of the number of older persons aged 65 years and older to the number of young persons (from 0 to 14 years old) [69]. In addition, despite the privileged latitude of Portugal, being the third European country with higher availability of solar radiation, followed by Cyprus and Malta [70], a high prevalence of vitamin D deficiency in older adults was recently reported [36]. Considering these facts, the association found in the present study between vitamin D deficiency and lower gait speed and hand grip strength values, reinforces the need to define strategies for the improvement of vitamin D status in Portuguese older adults.

The limitations were inherent of this study’s cross-sectional design that did not allow to conclude about causal inferences of vitamin D on gait speed and on hand grip strength. Although results were adjusted for a considerable set of factors, residual confounding could not be ignored. In relation to the laboratory analysis of serum 25(OH)D, the Vitamin D External Quality Assessment Scheme has revealed method-related differences in 25-(OH)D results, raising concerns about the comparability and accuracy of different assays [71, 72]. The competitive protein binding assay used in the present study was reported as very suitable for automated measurement of 25(OH)D [73], however, a potential overestimative of values should also not be ruled out [73]. Considering this fact, the low 25(OH)D levels found in the present study may be even more concerning, but it is not expected that the direction or strength of the observed associations are affected, since it reflects in a systematic overestimation.

In conclusion, in older adults, particularly in men, the risk of vitamin D deficiency was directly associated with the lowest values of gait speed and of hand grip strength. However, randomized controlled trials are needed to overcome the possibility of reverse causation and residual confounding. Present results emphasise the need for strategies to promote the reduction of the high prevalence of low vitamin D status among the Portuguese older adult population.
